# A Comparison of Er:YAG Laser with Photon-Initiated Photoacoustic Streaming, Nd:YAG Laser, and Conventional Irrigation on the Eradication of Root Dentinal Tubule Infection by* Enterococcus faecalis* Biofilms: A Scanning Electron Microscopy Study

**DOI:** 10.1155/2017/6215482

**Published:** 2017-11-27

**Authors:** Burcu Ozses Ozkaya, Kamran Gulsahi, Mete Ungor, Julide Sedef Gocmen

**Affiliations:** ^1^Oral and Dental Health Center, Hatay, Turkey; ^2^Department of Endodontics, Faculty of Dentistry, Başkent University, Ankara, Turkey; ^3^Department of Medical Microbiology, Faculty of Medicine, Başkent University, Ankara, Turkey

## Abstract

This study evaluated the antimicrobial efficacy of Er:YAG laser activation with photon-initiated photoacoustic streaming (PIPS), Nd:YAG laser disinfection, and conventional irrigation on* Enterococcus faecalis* biofilms using scanning electron microscopy (SEM). Biofilms were grown on 110 root halves and divided into the following: Groups 1 and 2 (saline and 1% NaOCl with apical position of PIPS, resp.), Groups 3 and 4 (saline and 1% NaOCl with coronal position of PIPS, resp.), Groups 5 and 6 (Nd:YAG laser after saline and 1% NaOCl irrigation, resp.) and Groups 7, 8, and 9 (conventional irrigation with 1% NaOCl, 6% NaOCl, and saline, resp.). SEM images of the apical, middle, and coronal levels were examined using a scoring system. Score differences between Groups 1 and 2 were insignificant at all levels in the remaining biofilm. Group 4 had significantly greater bacterial elimination than Group 3 at all levels. Differences in Nd:YAG laser irradiation between Groups 5 and 6 were insignificant. Groups 7 and 8 were insignificantly different, except at the middle level. Saline group had a higher percentage of biofilms than the others. In this study, PIPS activation with NaOCl eliminates more* E. faecalis* biofilms in all root canals regardless of the position of the fiber tip.

## 1. Introduction

Endodontic therapy disinfects the root canal and its three-dimensional tubular network [1]. Insufficient eradication of intraradicular bacteria results from the complex morphology of the root canal and the organization of intracanal bacteria into biofilms [2]. Biofilm bacteria are resistant to many antimicrobial agents used in endodontics [3]. Approximately 40%–60% of canals contain cultivable bacteria after sodium hypochlorite (NaOCl) syringe/needle irrigation and instrumentation [4, 5]. Irrigation is important for successful root canal treatment. Therefore, irrigant activation techniques have been proposed to improve irrigant distribution in the canal system and increase irrigation effectiveness [6, 7]. Irrigants used for this goal are NaOCl, chlorhexidine gluconate (CHX), and ethylenediaminetetraacetic acid (EDTA).

The neodymium-doped yttrium aluminum garnet (Nd:YAG) laser is useful for removing the smear layer and debris and for disinfection. The bactericidal efficacy of the Nd:YAG laser ranges from 77% to 86% at 10 Hz for 15 s [8] and from 97% to 99% at 200 mJ for 20 s [9] and is >99% at 1.5 W for 5 s [10].

Lasers activate irrigation solutions by transferring pulsed energy. The erbium-doped yttrium aluminum garnet (Er:YAG) laser wavelength (2940 nm) has the highest absorption in water and high affinity for hydroxyapatite, which makes it suitable for use in root canal treatment [11–13]. The study [13] reported that debriding and the cleaning efficacy of irrigation can be enhanced by a new erbium laser technique, which uses photon-induced photoacoustic streaming (PIPS) of irrigants produced by a newly designed tapered and stripped tip with specific minimally ablative laser setting comprising low energy (20 mJ), a pulse repetition rate of 15 Hz, and very short pulse duration (50 *μ*s). The laser tip is placed into the coronal access opening of the pulp chamber only and is kept stationary without advancing it into the orifice of the canal. By placing the tip into the coronal portion, PIPS can theoretically travel three-dimensionally to wherever fluid exists in the root canal and effectively debride the entire root canal system [14]. This* in vitro* study aimed to evaluate the antimicrobial efficacy of Er:YAG laser activation with PIPS, Nd:YAG laser disinfection, and conventional irrigation on intraradicular* E. faecalis* biofilms.

## 2. Materials and Methods

This study was approved by Başkent University Institutional Review Board (project number: D-DA11/05). 110 extracted single-rooted human teeth were individually autoclaved at 121°C for 15 min. Their crowns were removed and root lengths were standardized to 16 mm. The apical patency of each canal was established using a size #10 stainless steel K-file (VDW, Antaeos, Germany). The working length was 1.0 mm shorter than the actual root canal length. Sizes 5, 4, and 3 Gates-Glidden burs were used to flare the coronal aspect of each canal. The root canals were prepared by an F3 ProTaper rotary file (Dentsply Maillefer, Ballaigues, Switzerland). Each canal was instrumented up to a size 50 NiTi K-file (Dentsply Maillefer). Copious 2.5% NaOCl irrigation was used throughout the instrumentation. Each prepared root was placed into a block of silicone impression material (Zetaplus; Zhermack, Marl, Germany) within a plastic tray containing 12 plastic specimen jars, which ensured that the coronal end of the prepared root was flush with the surface of the silicone block.

After the impression material set, each root and corresponding silicone block were numbered and later matched. Each root was sectioned longitudinally through the root canal using a diamond disc and chisel. The most uniform half of each canal was selected for biofilm growth. To ensure good reapproximation, the root halves were placed back into their corresponding silicone index. The silicone block and the chosen root half were marked to ensure the correct orientation of each root in its block. The root halves were then removed from the silicone indices and immersed in 17% EDTA solution for 1 min to remove the smear layer and then washed thoroughly with water. The root halves were autoclaved at 121°C for 15 min. They were thereafter used for biofilm growth and stored at 100% humidity in Eppendorf tubes.

### 2.1. Preparation of Bacterial Inoculum

Biofilm formation was evaluated in sterile screw cap cell culture flasks (Greiner Bio-One GmbH, Frickenhausen, Germany) to minimize contamination.* Enterococcus faecalis* (ATCC 29212) was inoculated on blood agar and incubated at 37°C under microaerophilic conditions for 24 h. A bacteria suspension was prepared, based on the 0.5 McFarland (PhoenixSpec, BD, NJ, USA) turbidity standard, and diluted as 1/20.

### 2.2. Biofilm Formation

One-half of each root selected for biofilm growth was placed in separate cell culture flasks. Each half-root was immersed in 8 mL brain heart infusion (BHI) broth and bacteria suspension in cell culture flasks. The flasks were incubated at 37°C for 4 weeks until the biofilm formation matured. The BHI broth was changed weekly, and inoculated on blood agar for contamination. After biofilm formation, two teeth were randomly selected for scanning electron microscopy (SEM) imaging to confirm the formation of intraradicular* E. faecalis* biofilms and 1000 *μ*m depth of the tubule invasion by* E. faecalis*. 108 root-half pairs were reapproximated and randomly divided into nine groups of 12 samples each as follows:

  Group 1: the root canals were irrigated by conventional syringe irrigation with 5 mL of 0.9% saline solution for 40 s. Activation by PIPS laser was applied with the fiber tip at the apical level for 20 s and the irrigant was constantly deposited in the canal by 27-gauge needle. The solution was activated by an Er:YAG laser with 2940 nm wavelength (Fidelis Plus III AT; Fotona, Ljubljana, Slovenia) with a 14 mm long 400 *μ*m endodontic fiber. The laser operating parameters were 15 Hz, 35 mJ per pulse, and 50 *μ*s pulse duration. The coaxial water spray feature of the handpiece was off. The tip was inserted 5 mm short of the working length and activated for 20 s. A rubber stop was placed on the endodontic fiber at a length of 10 mm from the tip.
  Group 2: the same protocol as in Group 1, but 1% NaOCl solution was the irrigant.
  Group 3: the same protocol as in Group 1, but the PIPS fiber tip was applied at the coronal level.
  Group 4: the same protocol as in Group 3, but 1% NaOCl solution was the irrigant.
  Group 5: the root canals were irrigated by conventional syringe irrigation with 5 mL of 0.9% saline solution for 40 s. The specimens were dehydrated by paper points. Irradiation by Nd:YAG laser (Fidelis Plus III AT; Fotona, Ljubljana, Slovenia) at 1064 nm was administered at a power of 1.5 W and a frequency of 15 Hz. The optical fiber tip (200 *μ*m diameter) was inserted into the root canal 1 mm short of the working length and gently withdrawn spirally with the laser activated for 5 s; this procedure was repeated four times with a 15 s interval (i.e., the manufacturer's laser irradiation protocols).
  Group 6: the same protocol as in Group 5, but 1% NaOCl solution was the irrigant.
 
Table 1 shows irradiation parameters for Er:YAG and Nd:YAG lasers.
  Group 7: conventional syringe irrigation with 5 mL of 1% NaOCl solution was administered with a 27-gauge side-venting irrigating needle (Hayat, İstanbul, Turkey) for 60 s. A rubber stop was placed on each irrigating needle at a length of 15 mm from the tip. The needle was moved back and forth gently in the canal without binding.
  Group 8: the same protocol as in Group 7, but 6% NaOCl solution was the irrigant (i.e., negative control).
  Group 9: the same protocol as in Group 7, but 0.9% saline solution was the irrigant (i.e., positive control).

The remaining intracanal NaOCl was neutralized with sodium thiosulfate for 1 min.

### 2.3. Preparation and Examination by SEM

After the irrigation protocols, the samples were immediately washed three times with 0.1 M Sorensen's phosphate-buffered solution (PBS) and immersed in 2.5% phosphate-buffered glutaraldehyde fixative for 24 h at 4°C. The fixation samples were washed three times with 0.1 M PBS. The samples were then immersed in ascending grades of ethanol for 15 min at successive concentrations of 20%, 40%, 60%, 80%, and 90% and two cycles of 100%. The samples were dehydrated by a critical point dryer (model 815B; Tousimis, Rockville, MD, USA), coated with 80 *μ*m of gold-palladium by a precision etching coating system (model 682; Gatan, Warrendale, PA, USA), and observed under SEM (Quanta 200 F; FEI Company, Eindhoven, Netherlands).

### 2.4. Scanning Electron Microscope Observations

The root canals of each specimen were individually examined at the coronal, middle, and apical levels (4 mm, 7 mm, and 10 mm, resp., from the orifice). The same operator took 324 standard images (magnification, 700x). Three blinded observers (one specialist endodontist and two endodontic postgraduates) evaluated the SEM images using a scoring method to assess the remaining biofilm coverage [15]. The scoring index scores were as follows: “1,” less than 5% biofilm coverage; “2,” 5%–33% biofilm coverage; “3,” 34%–66% biofilm coverage; and “4,” 67%–100% biofilm coverage of the root canal wall.

### 2.5. Data Analysis

The data analyses were conducted using SPSS 15.0 software (SPSS Inc., Chicago, IL, USA). Differences between the experimental groups at each level were assessed using Pearson chi-squared analysis. The significance level was *p* < 0.05. Inter- and intraexaminer agreements were assessed by* kappa* analysis.

## 3. Results

**Figure 1:**
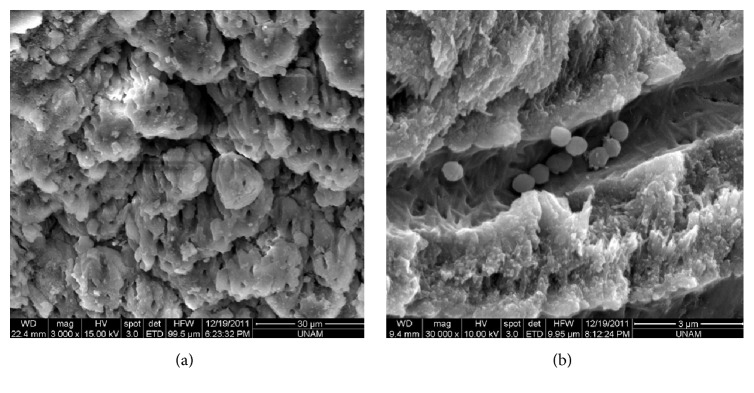
Scanning electron microscope images of biofilm formation. (a) Colonization of* Enterococcus faecalis* and a biofilm-like structure on the canal surface after 4 weeks of incubation (magnification, 3000x). (b)* Enterococcus faecalis* invasion into the dentin tubules (magnification, 30,000x).

**Figure 2:**
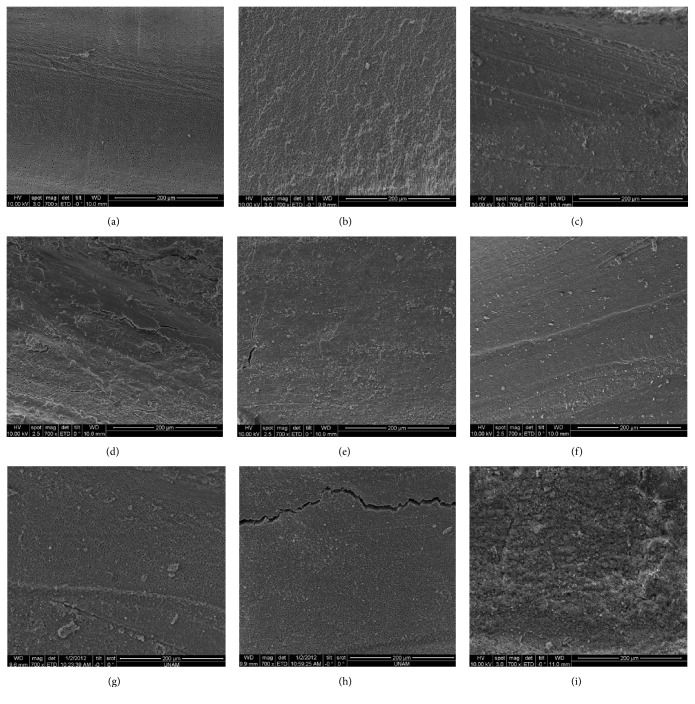
Scanning electron microscope analysis of the root canal surface after irrigation protocols at the apical levels of the root. Activation by PIPS laser at the apical level with (a) saline (Group 1) and (b) 1% NaOCl (Group 2). Activation by PIPS laser at the coronal level with (c) saline (Group 3) and (d) 1% NaOCl (Group 4). Irradiation by Nd:YAG laser after conventional irrigation (CI) with (e) saline (Group 5) and (f) 1% NaOCl (Group 6). The CI protocol with (g) 1% NaOCl (Group 7) and (h) 6% NaOCl (Group 8, the negative control). (i) The CI protocol with saline (Group 9, the positive control).

Figure 1 shows the SEM images of the incubation and* E. Faecalis* penetration into the dentin tubules.  Table 2 shows the quantitative evaluation of biofilm removal in the experimental groups. Groups 1 and 3 were significantly different at the middle and apical levels of the root canal (*p* < 0.05). Biofilm elimination was greater with apical positioning of the PIPS tip with saline activation (i.e., Group 1) than with coronal positioning of PIPS tip with saline activation (i.e., Group 3). The scores for the remaining biofilm coverage were insignificantly different between Groups 2 and 4 at all levels (i.e., apical and coronal PIPS tip position, resp., with 1% NaOCl solution activation) (*p* > 0.05). The scores were insignificantly different for the remaining biofilm coverage between Groups 1 and 2 (i.e., apical position of PIPS; Figures 2(a) and 2(b), resp.) at all levels (*p* > 0.05). Groups 3 and 4 (i.e., coronal position of PIPS) were significantly different at all levels. Group 4 (Figure 2(d)) had significantly greater bacterial elimination than Group 3 (Figure 2(c)) (*p* < 0.05). Insignificant differences existed between the Nd:YAG laser irradiation groups (i.e., Groups 5 and 6; Figures 2(e) and 2(f), resp.) at all levels (*p* > 0.05). Differences between Groups 7 and 8 (Figures 2(g) and 2(h), resp.) were insignificant at the apical and coronal levels but were significant at the middle part. Group 8, the negative control (6% NaOCl solution), had greater biofilm reduction at the middle level compared to Group 7 (1% NaOCl solution) (*p* < 0.05). Group 9, the positive control (0.9% saline solution), had a higher percentage of intraradicular* E. faecalis *biofilms at all levels compared to the other groups (Figure 2(i)).

At the coronal level, Groups 1, 3, and 5 were insignificantly different; however, biofilm elimination efficacy was less in Group 5 (Nd:YAG + saline) than in Group 1 (apical PIPS + saline) but was greater than that in Group 3 (coronal PIPS + saline) at the apical and middle parts of the canal (i.e., bacterial reduction was Group 1 > Group 5 > Group 3). Scores were insignificantly different for the remaining biofilm coverage at all levels between Group 6 (Nd:YAG + 1% NaOCl), Group 2, and Group 4 (apical and coronal PIPS position, resp., with 1% NaOCl solution activation) (*p* > 0.05). However, these groups (Groups 2, 4, and 6) had greater biofilm reduction at all levels compared to Group 7 (1% NaOCl solution). The* kappa* values for intraobserver and interobserver agreement were >0.75 and >0.53, respectively.

## 4. Discussion

This* in vitro* study used SEM to compare the antimicrobial efficiency of Er:YAG laser activation with PIPS, Nd:YAG laser irradiation, and conventional irrigation on* E. faecalis* biofilms at different root canal levels.* Enterococcus faecalis* is well studied because of its high virulence, penetration into dentinal tubules, and adherence to collagen, and it is the most frequently isolated microorganism in endodontic treatment failure [1, 16, 17]. The biofilm formation time differs between studies, but resistance to antimicrobial therapy is higher in 3-week-old (i.e., mature) bacterial biofilms than in young biofilms [18]. In the current study, the incubation period was 4 weeks to enhance bacterial penetration of* E. faecalis* into the dentin tubules and promote bacterial biofilm formation.

The Nd:YAG laser exhibits bactericidal effects on dentin up to a depth of 1000 *μ*m. This laser also effectively removes debris and treats apical inflammation [1]. The energy of Er:YAG laser is rapidly absorbed and eliminated in biofilms on dental hard tissues because of the water content of biofilms and because Er:YAG laser light absorption by water is high [19, 20]. Laser energy kills bacteria directly and activates the irrigant to enhance its bactericidal actions. The mechanism of laser-activated irrigation may depend on rapid fluid motion caused by the expansion and implosion of laser-induced bubbles. The implosion impacts the root canal surfaces, thereby causing shear forces, surface deformation, and removal of the surface material [21]. A novel laser agitation irrigation technique, photon-initiated photoacoustic streaming, is capable of disinfecting, cleaning, and debriding the root canal system after instrumentation, even with sterile water activated by a photomechanical effect [13, 14].

This study used an Er:YAG laser with a newly designed radial quartz and stripped tip. The wavelength of the Er:YAG laser was 2940 nm to produce effective activation and streaming of fluids within the canal.

As Table 2 shows, in the PIPS system + saline groups (i.e., Groups 1 and 3), apical positioning of the fiber tip more greatly reduced the bacterial biofilms in the middle and apical levels compared to coronal positioning. The antimicrobial efficacy of activated saline solution was the same at the coronal levels regardless of the fiber tip position. This result agrees with the findings of previous studies [6, 22–24]. When lasers are used for irrigant activation, the most intense fluid motion is near the fiber tip; therefore, positioning the tip deeper inside the canal may eliminate the biofilm more greatly than inserting the tip into the canal orifice. Biofilm reduction was the same at all levels when the PIPS system was used with 1% NaOCl (i.e., Groups 2 and 4) regardless of the fiber tip position. Laser treatment may significantly increase the chemical action of NaOCl [6].

Penetration of the laser tip is another factor in eradicating biofilm formation. The current study showed that applying PIPS apically (i.e., Groups 1 and 2) similarly reduced the biofilm at the apical, middle, and coronal root canal levels, regardless of whether saline or NaOCl was used [24, 25]. However, when applying PIPS at the canal entrance, NaOCl more effectively eliminated the biofilm than saline (i.e., Groups 4 and 3, resp.) at all root levels. This finding could be attributed to the enhanced bactericidal, dissolution, and cleaning effects of NaOCl when activated by laser energy. This result also agrees with those of previous studies [11, 22, 25–27].

Biofilm removals were similar in the Nd:YAG laser irradiation groups (i.e., Groups 5 and 6) at all root levels. The antibacterial effect of Nd:YAG laser results from the absorption of the laser light into dentin and it induces bacterial death [1]. Biofilm reduction with conventional irrigation was greater with 6% NaOCl than with 1% NaOCl (Groups 8 and 7, resp.; *p* < 0.05) at the middle level. Biofilm elimination was the same at the apical and coronal levels because of the needle tip position in the canal and the NaOCl concentration. This finding may be because the irrigant first contacts the middle area with higher concentration of NaOCl and thereby causes greater biofilm reduction at this level.

Traditional syringe irrigation is ineffective at the apical portion of the root canal because it delivers solutions no further than 1 mm past the tip of the needle [28]. In a closed canal system, irrigant extrusion beyond 1–1.5 mm of the needle could generate a liquid film along the air bubble-canal wall interface [29].

A high concentration of NaOCl has a better effect than a low concentration [30]. Contact with a biofilm, organic tissue, and hard tissue (i.e., dentin) reduced the effect of NaOCl; therefore, the effect of an irrigant could be decreased in the apical and coronal area. One study [15] showed that an* E. faecalis* biofilm can be completely dissolved using 6 mL of 1% NaOCl for 2 min. This result differs from our findings, which can be attributed to the irrigation time (e.g., a 1 min irrigation time was applied).

Biofilm removals by two different laser systems using saline (i.e., Groups 1, 3, and 5) were similar at the coronal level of the root. However, Nd:YAG had less biofilm elimination efficacy than apical positioning of PIPS but had greater elimination efficacy compared to coronal positioning of PIPS at the apical and middle parts of the canal. This result could be related to the fiber tip position. Laser energy can eliminate the bacterial structure; therefore, biofilm reduction is the same at the coronal level of the root. Other studies demonstrated that bacterial reduction with Er:YAG laser was greater than [20, 31] or the same [1] as that of the Nd:YAG laser. These differences may be attributable to the irradiation time and output condition of the laser, incubation period of the biofilm, irrigation solutions, test models, and evaluation methods.

However, applying PIPS (i.e., apical or coronal positioning of the fiber tip) and Nd:YAG laser with 1% NaOCl solution (i.e., Groups 2, 4, and 6) was similarly effective for biofilm reduction at the apical, middle, and coronal levels of the canal (*p* > 0.05). This finding could be related to using NaOCl. These groups (i.e., Groups 2, 4, and 6) had greater biofilm reduction at all levels compared to Group 7 (conventional irrigation with 1% NaOCl solution). Laser treatment seems to significantly increase the antimicrobial efficacy of NaOCl.

## 5. Conclusions

In this* in vitro* study, the irrigation solution type and fiber tip position were important. Activation of NaOCl by PIPS laser can eliminate* E. faecalis* biofilm at all root canal levels regardless of the fiber tip position. The Nd:YAG laser achieved the same result. Activation by PIPS laser in the apical position can significantly reduce* E. faecalis* biofilms at the apical and middle levels compared to Nd:YAG laser irradiation when using saline solution.

## Figures and Tables

**Table 1:** Irradiation parameters.

Parameter	Source type		Parameter unit (metric or SI)
	Er:YAG	Nd:YAG	
Wavelength	2940	1064	Nm
Spot size	400	200	*µ*m
Operating frequency	15	15	Hz
Pulse energy	35	-	mJ
Pulse width	50	-	*µ*s
Power	0.5	1.5	W
Energy density	6.963	-	J/cm^2^
Power density	99.471	1193	W/cm^2^

**Table 2:** Quantitative evaluation of biofilm reduction. Mean ± standard deviation, median, and minimum and maximum values of biofilm elimination after root canal irrigation for each group.

Group	Apical		Middle		Coronal	
	Mean ± SD	Median (min-max)	Mean ± SD	Median (min-max)	Mean ± SD	Median (min-max)
1	1.00 ± 0.0	1 (1-1)	1.08 ± 0.3	1 (1-2)	2.08 ± 1.1	2 (1-4)
2	1.25 ± 0.5	1 (1-2)	1.42 ± 0.7	1 (1-3)	1.42 ± 0.7	1 (1-3)
3	2.92 ± 0.7	3 (2-4)	2.58 ± 0.8	3 (1-4)	2.17 ± 0.6	2 (1-3)
4	1.67 ± 0.7	2 (1-3)	1.50 ± 0.7	1 (1-3)	1.08 ± 0.3	1 (1-2)
5	1.50 ± 0.5	1.5 (1-2)	1.67 ± 0.5	2 (1-2)	1.92 ± 0.8	2 (1-3)
6	1.58 ± 0.5	2 (1-2)	1.75 ± 0.6	2 (1-3)	1.83 ± 0.7	2 (1-3)
7	2.17 ± 0.6	2 (1-3)	2.50 ± 0.9	3 (1-4)	2.00 ± 1.0	2 (1-4)
8	2.17 ± 0.4	2 (2-3)	2.00 ± 0.4	2 (1-3)	1.92 ± 0.7	2 (1-3)
9	3.70 ± 0.5	4 (3-4)	3.50 ± 0.5	3.5 (3-4)	3.40 ± 0.7	3.5 (2-4)

Groups 1 and 2: saline and 1% NaOCl with apical position of PIPS, respectively; Groups 3 and 4: saline and 1% NaOCl with coronal position of PIPS, respectively; Groups 5 and 6: Nd:YAG laser after conventional irrigation with saline and with 1% NaOCl, respectively; Groups 7 and 8: conventional irrigation with 1% NaOCl and with 6% NaOCl, respectively, negative control; and Group 9: saline solution, positive control).
